# The Yeast PCNA Unloader Elg1 RFC-Like Complex Plays a Role in Eliciting the DNA Damage Checkpoint

**DOI:** 10.1128/mBio.01159-19

**Published:** 2019-06-11

**Authors:** Soumitra Sau, Batia Liefshitz, Martin Kupiec

**Affiliations:** a School of Molecular Cell Biology and Biotechnology, Tel Aviv University, Ramat Aviv, Israel; Tel Aviv University; Bar Ilan University; Hebrew University of Jerusalem

**Keywords:** DNA damage checkpoint, DNA repair, DNA replication, Dpb11, PCNA, RFC-like complex, Rad9, *Saccharomyces cerevisiae*, yeast

## Abstract

The PCNA (proliferating cell nuclear antigen) ring plays central roles during DNA replication and repair. The yeast Elg1 RFC-like complex (RLC) is the principal unloader of chromatin-bound PCNA and thus plays a central role in maintaining genome stability. Here we identify a role for Elg1 in the unloading of PCNA during DNA damage. Using DNA damage checkpoint (DC)-inducible and replication checkpoint (RC)-inducible strains, we show that Elg1 is essential for eliciting the signal in the DC branch. In the absence of Elg1 activity, the Rad9 (53BP1) and Dpb11 (TopBP1) adaptor proteins are recruited but fail to be phosphorylated by Mec1 (ATR), resulting in a lack of checkpoint activation. The chromatin immunoprecipitation of PCNA at the Lac operator sites reveals that accumulated local PCNA influences the checkpoint activation process in *elg1* mutants. Our data suggest that Elg1 participates in a mechanism that may coordinate PCNA unloading during DNA repair with DNA damage checkpoint induction.

## Abstract (precis)

The Elg1protein forms an RFC-like complex in charge of unloading PCNA from chromatin during DNA replication and repair. Mutations in the *ELG1* gene caused genomic instability in all organisms tested and cancer in mammals. Here we show that Elg1 plays a role in the induction of the DNA damage checkpoint, a cellular response to DNA damage. We show that this defect is due to a defect in the signal amplification process during induction. Thus, cells coordinate the cell's response and the PCNA unloading through the activity of Elg1.

## INTRODUCTION

A stable genome is needed to ensure the accurate transmission of the genetic material from one cell division to the next. Genome stability is constantly compromised by the normal metabolism of the cell, as well as by external insults, which can pose a threat to viability (1) or, in mammalian cells, can lead to the development of cancer (2). The DNA is more prone to chemical modifications and breakage during DNA replication or repair, when the DNA double helix must be opened (3, 4). Most spontaneous chromosomal rearrangements and mutations probably arise in these situations (5–7). During the process of DNA replication, the activity of the DNA polymerases may be impaired by the presence of DNA lesions, bound proteins, and secondary structures and by collisions with other DNA-interacting proteins such as RNA polymerases or topoisomerases (8–11). These encounters may lead to stalling or even collapse of replication forks, creating double-strand breaks (DSBs), or to repriming events that leave single-strand DNA gaps behind the fork (12–14).

The presence of DNA damage or stalled replication forks elicits an organized response, orchestrated by conserved protein kinases, which respond to the threat by arresting cell cycle progression, inducing DNA repair or lesion bypass mechanisms, and, when necessary, restoring DNA replication (15, 16). This DNA damage response (DDR) checkpoint mechanism has been shown to be divided into two branches in all organisms studied. The DNA checkpoint (DC) responds to double-stranded breaks (DSBs) or single-stranded DNA (ssDNA) gaps left behind by the replicating machinery (13, 17). In the yeast Saccharomyces cerevisiae, the presence of RPA-coated ssDNA acts as a signal to recruit sensor kinase Mec1 (ATR in mammals) and its binding partner Ddc2 (ATRIP) (18); at the same time, the 9-1-1 clamp, a PCNA-like molecule, is loaded independently by the Rad24 RFC-like complex (RLC) (mammalian Rad17) at the junction between RPA-coated single-stranded DNA and double-stranded DNA (19). Once in place, the Ddc1 subunit of the 9-1-1 clamp helps to recruit Dpb11 (TopBP1 in mammals) (20), increasing the activity of Mec1, which phosphorylates the nearby histone H2A. Both phosphorylated H2A and activated Dpb11 promote the recruitment of Rad9 (mammalian 53BP1) (20), which undergoes phosphorylation by Mec1. Phosphorylated Rad9 anchors the checkpoint effector Rad53 kinase (Chk2 in mammals) and allows Mec1-dependent phosphorylation, which facilitates autophosphorylation and activation of Rad53 (21). This hierarchical activation of protein kinases ensures amplification of the local checkpoint signal before a more global response, which includes the phosphorylation of hundreds of target proteins, takes place (22). An additional major kinase, Tel1 (ATM in mammals), can sometimes replace Mec1 in the activation of Rad53 (reviewed in reference 23).

A second branch of the DNA damage response is the replication checkpoint (RC). In response to stalled replication forks encountered during the S phase, the cells activate Mec1 and Rad53 but use the Mrc1 (mammalian claspin) adaptor protein in place of Rad9 (24, 25). Recently, it was shown that the DC and the RC also differ in the location of the inducing lesion: whereas the Mrc1-mediated RC is located at the stalled fork, the Rad9-mediated DC probably acts behind the fork, in ssDNA gaps left by repriming (13).

The ultimate goal of both branches of the DNA damage checkpoint is that of allowing the cells to either repair the damage or bypass it in a way that allows cell cycle progression (26, 27). A central molecule in DNA repair and replication is the homotrimeric ring PCNA. It plays a dual role as a processivity factor for DNA polymerases and a “landing pad” for additional proteins (28, 29). In addition, PCNA serves as a signaling platform, as it undergoes posttranslational modifications, such as ubiquitination and SUMOylation, which determine the repair/tolerance pathway that the cells eventually take. PCNA is loaded onto DNA by the RFC complex, composed of a large subunit (Rfc1) and four small subunits (Rfc2 to Rfc5); a similar complex, in which the conserved Elg1 protein replaces the Rfc1 subunit, is in charge of unloading it (reviewed in reference 30).

Although it is not essential for life, deletion of *ELG1* results in gross chromosomal rearrangements, increased sensitivity to genotoxic agents, elevated rates of spontaneous recombination and chromosome loss, loss of sister chromatid cohesion, and elongated telomeres (31). The Elg1 RFC-like complex (RLC) is able to unload both SUMOylated and un-SUMOylated forms of PCNA from the chromatin in yeast (32–35) and is able to unload both ubiquitinated or unmodified PCNA forms in human cells (36). The mouse ortholog of *ELG1* (ATAD5) is essential for life and acts as a tumor suppressor (37). The human ortholog participates in the Fanconi anemia pathway (38, 39), and mutations in this gene are associated with different types of human cancer (37, 40).

Yeast Elg1 has been shown to undergo phosphorylation in a Mec1-dependent fashion following exposure to DNA-damaging agents (41). Here we show that the Elg1 protein plays a role in the induction of the DNA checkpoint (DC) branch of the DDR. This, in principle, provides a way of coordinating PCNA unloading with checkpoint activation.

## RESULTS

### PCNA unloading after DNA damage requires Elg1 phosphorylation.

We have previously shown that upon exposure to DNA damage, Elg1 undergoes phosphorylation at a serine (S) at position 112 of the protein. This serine is followed by glutamine (Q), forming an SQ motif, representing the preferred phosphorylation sites for the apical kinases ATM^Tel1^ and ATR^Mec1^ (41). In addition to this site, serines 6 and 8 have also been shown by mass spectrometry analysis to undergo phosphorylation (41). The primary function of the Elg1–replication factor C-like complex (Elg1-RLC) is to unload chromatin-bound PCNA (32, 42). As all the cellular abnormalities that are due to the absence of Elg1 that have been identified so far have been attributed to its chromatin-bound PCNA unloading function, we sought to understand the biological relevance of the Elg1 phosphorylation in the context of PCNA unloading. To probe the PCNA unloading activity in the absence of Elg1 phosphorylation, we performed chromatin fractionation assays with several phosphorylation-deficient (phospho-deficient) *elg1* mutants in which serines at positions 6, 8, and 112 were changed either to alanine (A), a small, neutral amino acid, or to glutamic acid (E), a positively charged residue that often, although not always, mimics a phosphorylated state (41).

**FIG 1:**
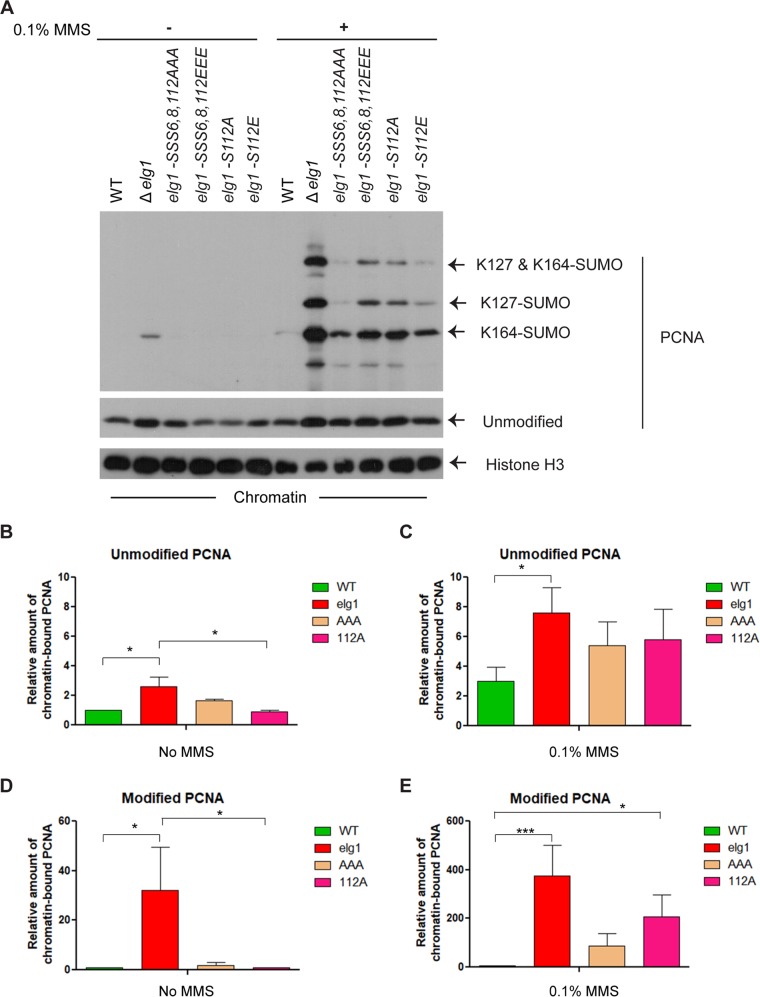
Absence of Elg1 phosphorylation causes PCNA accumulation on chromatin under conditions of DNA damage. One half of the logarithmically growing wild-type, *Δelg1*, and several Elg1 phospho-deficient mutant cells were treated with 0.1% MMS for 1 h to induce DNA damage, while the other halves were left untreated. Following the treatment, a chromatin fractionation assay followed by Western blotting was performed to detect the PCNA retention on chromatin. (A) Western blot with anti-PCNA antibody showing the different levels of chromatin-bound unmodified and modified (SUMOlyated) PCNA. The histone H3 was used as a chromatin-bound control and also served as a loading control. (B to E) Quantitation of unmodified PCNA under conditions of no MMS treatment (B) and MMS treatment (C) and of modified PCNA under conditions of no MMS treatment (D) and MMS treatment (E). At least three independent experiments were done with results measured; error bars represent standard errors of the means (SEM). *P* values were obtained using one-way analysis of variance (ANOVA), and statistical significance tests of differences between groups were done using Tukey’s multiple-comparison test. *P* values for the data shown in panels B, C, D, and E are 0.0175, 0.0382, 0.0285, and <0.001, respectively. *, *P* < 0.05; ***, *P* < 0.001.

Logarithmically growing wild-type (WT), *Δelg1*, and phospho-deficient *elg1* mutant cells were either treated with 0.1% methyl methanesulfonate (MMS) for 1 h or left untreated and were then processed for chromatin fractionation assay as described in Materials and Methods. As previously seen, in the absence of DNA damage, both unmodified PCNA and SUMOylated PCNA accumulated on chromatin in *Δelg1* cells, whereas it was efficiently removed in WT cells (Fig. 1A to D; see also Fig. S1 in the supplemental material). In contrast, almost no accumulation was observed in the *elg1* phosphorylation mutants, indicating that they were proficient for PCNA unloading in undamaged chromatin (Fig. 1A, B, and D). After challenge of the cells with MMS for 1 h, *Δelg1* cells showed a robust accumulation of chromatin-bound unmodified PCNA, which was increased 2.5-fold compared to that of WT cells, and a dramatic (∼400-fold) increase in the level of accumulated SUMOylated PCNA (Fig. 1A, C, and E). Under those conditions, all the mutants defective for phosphorylation also showed an increase in chromatin-bound unmodified PCNA. The increase was slightly lower than that obtained in the total absence of Elg1, but both unmodified PCNA and SUMOylated PCNA accumulated (Fig. 1A, C, and E). For *elg1-S112E* and *elg1SSS6,8,112EEE* mutants, we hoped that changing the serine residue to a glutamine residue might produce a result mimicking the Elg1 phosphorylation state, but even these mutants showed an accumulation of both unmodified and modified chromatin-bound PCNA compared to the level seen with the WT strain (Fig. 1A). This suggests that for Elg1, either the conversion of residues S to E was not effective in mimicking the phosphorylation state or S112 plays an important structural role in unloading of chromatin-bound PCNA together with its phosphorylated form following DNA damage. In addition, mutation of the single S112 residue led to a defect in PCNA unloading similar to that seen with the triple mutants defective for phosphorylation of serines 6, 8, and 112 together. Taken together, these results suggest that S112 of Elg1 plays no important role in PCNA unloading during regular DNA replication but becomes important when cells are exposed to DNA damage.

10.1128/mBio.01159-19.1 FIG S1 Flow cytometry analysis of logarithmically growing WT, *Δelg1*, *elg1-S112A*, and *elg1SSS6,8,112AAA* cells used for the chromatin fractionation assay described in the Fig. 1 legend. Download FIG S1, TIF file, 0.3 MB. Copyright © 2019 Sau et al. 2019 Sau et al. This content is distributed under the terms of the Creative Commons Attribution 4.0 International license.

### Elg1 is required to activate Rad53 kinase in a checkpoint-induced DNA damage system.

**FIG 2:**
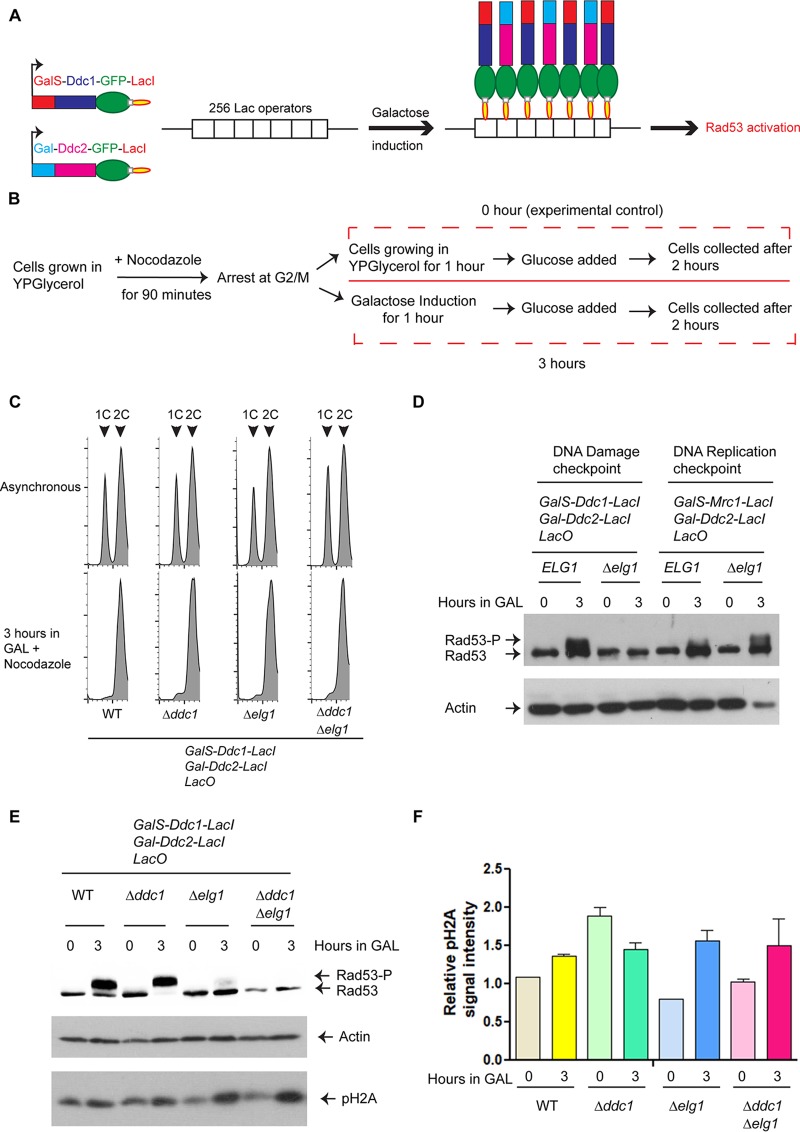
The Elg1-RLC is required for the DNA damage checkpoint activation. (A) A schematic representation of the system used. From each strain, two proteins (either Ddc1 and Ddc2 or Mrc1 and Ddc2) were expressed as fusions to the Lac repressor (LacI) from a galactose (Gal)-inducible promoter (GalS or Gal). Binding of these proteins to an array of 256 copies of the Lac operator (LacO) caused induction of either the DNA damage checkpoint (DC) or the DNA replication checkpoint (RC), which can be detected as phosphorylation of Rad53. For simplicity, only the DC is represented here. (B) The schematic diagram represents the experimental regimen. Briefly, cells carrying galactose-inducible checkpoint proteins fused to the Lac repressor and LacO_256_ constructs were grown in YP medium containing glycerol. At an OD_610_ of approximately 1.0, cells were arrested at G_2_-M by adding nocodazole (15 μg/ml) for 90 min and were then divided into two halves; one half received galactose (final concentration, 2%) to induce expression of *DDC1/MRC1* and *DDC2* for 1 h. For the same duration, growth of the other half continued in YP medium containing glycerol (which served as 0 h or the experimental control). After an hour, both halves received glucose (final concentration, 2%) and were allowed to grow for another 2 h before extraction of proteins. (C) Flow cytometry analysis of the WT, *Δddc1*, *Δelg1*, and *Δddc1 Δelg1* cells that were used for subsequent checkpoint induction experiments (see also Fig. S3). (D) Level of Rad53 phosphorylation in *ELG1* and *Δelg1* cells after induction of checkpoint fusions. (E) Level of Rad53 and histone H2A phosphorylation in WT, *Δddc1*, *Δelg1*, and *Δddc1 Δelg1* cells after induction of the checkpoint. (F) Quantitation of histone H2A phosphorylation intensity (relative to actin levels). At least three independent experiments were done and results measured; error bars represent standard errors of the means (SEM). Actin was used as a loading control for each Western blot.

S. cerevisiae *ELG1* is a conserved gene involved in many aspects of genome stability; deletion of the gene causes increased rates of spontaneous recombination, chromosome loss, gross chromosomal rearrangements, increased sensitivity to DNA-damaging agents, and elongated telomeres (33, 43–46). Our previous report on the Mec1-dependent phosphorylation of Elg1 following MMS-induced DNA damage (41) and the current observation on a role of Elg1 S112 in chromatin-bound PCNA unloading under the same conditions suggest a possible link between the Elg1-RLC activity and the DNA damage checkpoint. To separate potential roles of Elg1 in DNA repair from those in checkpoint induction, we took advantage of the elegant systems developed by Toczyski and colleagues (47, 48), in which either the DNA damage checkpoint (DC) or the replication checkpoint (RC) is initiated by recruiting galactose-induced proteins to an array of bacterial Lac operators in the absence of actual DNA damage or replication fork stalling (Fig. 2A). This system can thus identify a possible role of Elg1 in signaling, independent of a possible role of Elg1 in the repair of the DNA damage. In the first strain, Ddc1 (part of the 9-1-1 complex) and Ddc2 (partner of Mec1^ATR^), each fused to the Lac repressor protein, are recruited to the LacO array, eliciting a Rad9-dependent DNA damage response (47). In the second, the Mrc1 adaptor is coexpressed with Ddc2, eliciting the Rad9-independent replication checkpoint (48). The two responses are monitored by testing the phosphorylation level of the Rad53^CHK2^ kinase, a major checkpoint player that is among its first targets. Using these systems, we sought to answer the following two specific questions. (i) Does Elg1 play any role in the checkpoint induction process? (ii) Which of the two checkpoints is affected? We deleted *ELG1* in the two strains and performed checkpoint induction assays as described in the Fig. 2B legend. All the galactose induction assays were carried out with G_2_-M-arrested cells to avoid cell cycle complications (Fig. 2C) (47). We found that Elg1 was not required for the replication checkpoint pathway, as the phosphorylation of Rad53 seen in *Δelg1* cells was similar to that seen in the WT (Fig. 2D). In contrast, Elg1 was required to elicit DNA damage checkpoint pathway; phosphorylation of Rad53 was completely absent in *Δelg1* cells where Ddc1-green fluorescent protein-LacI (Ddc1-GFP-LacI) and Ddc2-GFP-LacI were coexpressed (Fig. 2D).

10.1128/mBio.01159-19.3 FIG S3 Flow cytometry analysis of asynchronous and alpha-factor-arrested cells of wt and *elg1* mutants after induction of the DNA damage checkpoint. Download FIG S3, TIF file, 0.4 MB. Copyright © 2019 Sau et al. 2019 Sau et al. This content is distributed under the terms of the Creative Commons Attribution 4.0 International license.

The strain inducing the DC (expressing Ddc1 and Ddc2) carries a single LacI-fused version of Ddc1 and is deleted for the natural *DDC1* gene. To rule out a possible artifact related to this fact, we repeated our experiment in a strain deleted for *ELG1* and carrying a normal copy of *DDC1.* We detected Rad53 phosphorylation in the WT strain and the *Δddc1* mutant, but phosphorylation of Rad53 was completely absent in the *Δelg1* and *Δelg1 Δddc1* strains (Fig. 2E). These results further confirmed that Elg1 is indeed required to activate the Rad53 kinase in a checkpoint- induced DNA damage system. As the *Δelg1* and *Δelg1 Δddc1* mutants behave similarly in the context of Rad53 phosphorylation, we have used these mutants interchangeably in this study.

### Recruitment of Ddc1-GFP-LacI and Ddc2-GFP-LacI to the Lac operator is unperturbed in a *Δelg1* mutant.

Previous work showed that recruitment of coexpressed Ddc1-GFP-LacI and Ddc2-GFP-LacI at the chromosomally integrated Lac operator sites is the crux of the system for activation of the Rad53 kinase, despite the fact that colocalization of these two induced checkpoint proteins does not create *de novo* DNA breaks (47). Once Ddc1 and Ddc2 are recruited, a large domain of the chromatin around the recruitment site becomes phosphorylated at serine 129 of the histone H2A protein, similarly to the phosphorylation of H2AX in mammals (49). Panels E and F of Fig. 2 show that a phospho-S129-specific antibody detected a signal after Ddc1 and Ddc2 induction in *Δelg1* and *Δelg1 Δddc1* cells that was indistinguishable from the results seen with WT cells. We therefore conclude that the absence of Rad53 phosphorylation in *Δelg1* cells is not due to lack of colocalization of Ddc1 and Ddc2 at the Lac operator sites.

### Ctf18 is dispensable for Rad53 activation.

**FIG 3:**
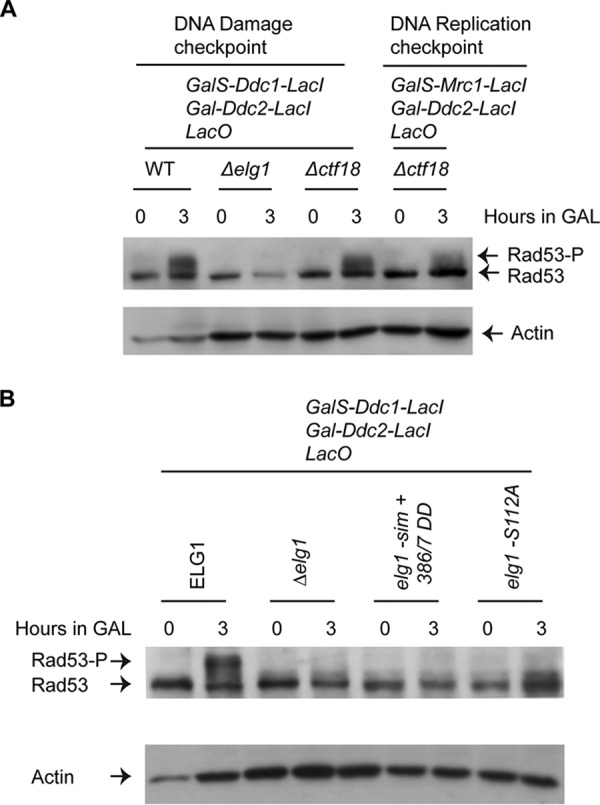
CTF18-RLC is not required for DNA damage checkpoint activation, whereas *elg1* mutants fail to activate Rad53 kinase. (A) Level of Rad53 phosphorylation in WT, *Δelg1*, *Δddc1 Δctf18*, and *Δctf18* cells after induction of both checkpoint fusions, as indicated. Actin was used as a loading control for each Western blot. (B) Level of Rad53 phosphorylation in *Δelg1*, *elg1-sim*+*386/7 DD*, and *elg1-S112A* cells after induction of Ddc1-GFP-LacI and Ddc2-GFP-LacI as described in the Fig. 2B legend. Actin was used as a loading control.

Like Elg1, Ctf18 interacts with four small Rfc subunits (Rfc2 to Rfc5) to form an RFC-like complex (RLC) (50, 51). Ctf18-RLC shows both loading and unloading of PCNA *in vitro* and is essential for the activation of the DNA replication checkpoint (52, 53). To address the role of Ctf18 in Rad53 activation in the checkpoint fusion system, we deleted *CTF18* in the strains with the inducible DNA damage and replication checkpoints. Panel A of Fig. 3 shows that deletion of *CTF18* had no effect. These results show that the Elg1-RLC, but not the Ctf18-RLC, is required for the activation of the DNA damage checkpoint.

### Functionally compromised Elg1 fails to activate Rad53 kinase.

In the absence of Elg1, or in the absence of phosphorylation at S112, PCNA accumulates on the chromatin in cells exposed to genotoxins (Fig. 1) (32, 33). Elg1 has been shown to interact physically with Rad53 (54). We have now observed that in the absence of Elg1, the Rad53 kinase is not activated by phosphorylation (Fig. 2). We thus sought to understand whether Rad53 phosphorylation requires (i) a physical interaction with the Elg1 protein, (ii) the chromatin-bound PCNA unloading function of Elg1, or (iii) the Mec1-dependent phosphorylation of Serine112 of Elg1. Our previous work showed that a mutant carrying mutations in Elg1's SUMO interaction motifs (SIMs) and substitutions of two threonines (T) at positions 386 and 387 of Elg1 to aspartic acid (D) (*elg1-sim+386/7DD*) was defective in PCNA unloading activity, despite the fact that the Elg1 protein was expressed at normal levels (42). We created derivatives of the strain with the inducible DNA damage checkpoint carrying either the *elg1-sim+386/7DD* allele or the *elg1-S112A* allele and performed experiments with these mutants along with WT and *Δelg1* cells as described in the Fig. 2B legend. Panel B of Fig. 3 shows that all three *elg1* mutants were defective in the phosphorylation of Rad53. This result shows that the mutants lacking both the Elg1 protein and Elg1’s activity (Δ*elg1*) or lacking only Elg1’s activity (*elg1-sim+386/7DD*) or mutated for serine 112 lacked Rad53 phosphorylation. We conclude that the activation of Rad53 is regulated by the chromatin-bound unloading function of Elg1 and possibly by its phosphorylation.

### Recruitment of Rad9 and Dpb11 at the Lac operator site is unperturbed in *Δelg1* mutants.

**FIG 4:**
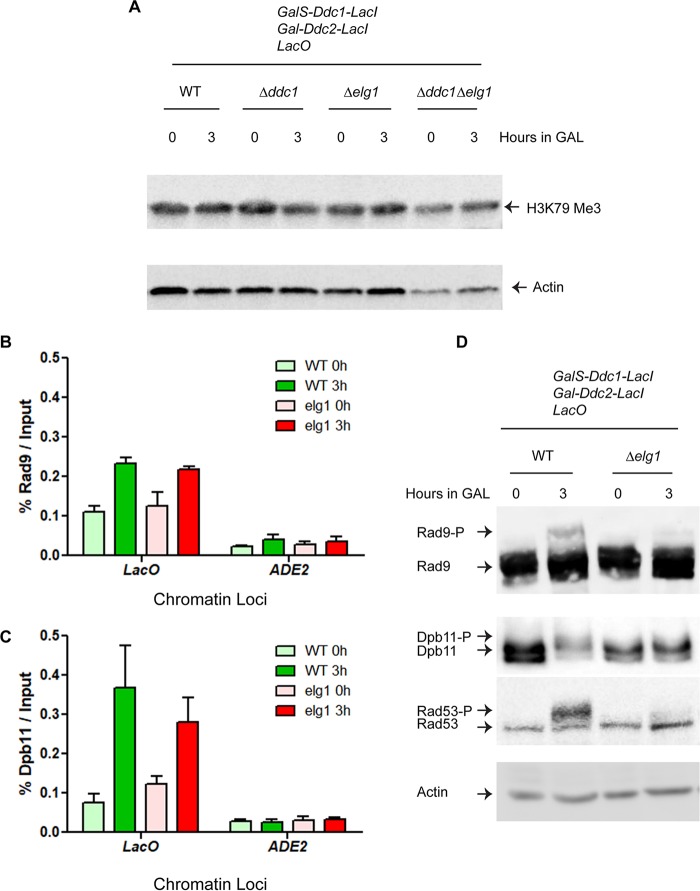
Absence of Elg1 inhibits the phosphorylation of Rad9 and Dpb11. (A) The level of histone H3 methylation at lysine residue 79 in the indicated strains. (B and C) The C termini of *RAD9* and *DPB11* were tagged with *FLAG* and *MYC* epitopes, respectively, in the checkpoint fusion strains (both WT and the *Δelg1* mutant). The checkpoint induction experiments were carried out as described in the Fig. 2B legend, and Rad9 and Dpb11 were pulled down using anti-FLAG and anti-Myc antibodies, respectively. The histograms of chromatin-immunoprecipitated Rad9 and Dpb11 represent the levels of recruitment of Rad9 (B) and Dpb11 (C) at the LacO array in WT and *Δelg1* cells. *ADE2* amplification represented the experimental control. The *LacO* and *ADE2* primers are listed in Table S2. At least three biological repeats were carried out; error bars represent standard errors of the means (SEM). *P* values were obtained using one-way ANOVA, and statistical significance tests of differences between groups were done using Tukey’s multiple-comparison test. *P* values for the data shown in Fig. 4B and C are 0.0067 and 0.0387, respectively. *ns*, not significant. (D) Phosphorylation levels of Rad9, Dpb11, and Rad53 in WT and *Δelg1* cells (the same cells as those used for the experiments whose results are presented in panels B and C). In the experiments whose results are presented in panels A and D, actin was used as a loading control.

In the DNA damage checkpoint pathway, Rad9 (53BP1 in mammals) acts as an adaptor/mediator protein that transduces the signal from activated Mec1 to the Rad53 effector kinase and also serves as a scaffold to promote Rad53 autophosphorylation (55). It collaborates tightly with Dpb11 (TopBP1), which is recruited to the damaged sites through its interaction with the phosphorylated Ddc1 subunit of the 9-1-1 complex, and becomes phosphorylated in a Mec1 kinase-dependent and Ddc1-dependent manner (56). Once recruited, Dpb11 promotes the full activation of Mec1 and the phosphorylation of Rad9 (20, 55). Thus, these two proteins work together to amplify the checkpoint signal. Recruitment of Rad9 to the chromatin, and, specifically, to damaged DNA sites, depends primarily on its interaction with (i) Dot1-mediated lysine (K) 79 methylated histone H3 and (ii) Mec1/Tel-mediated phosphorylated S129 of histone H2A (57). Since histone H2A S129 phosphorylation by Mec1 takes place normally in the absence of Elg1 activity (Fig. 2F), we measured the levels of histone H3 K79 methylation in *Δelg1* and WT cells. Panel A of Fig. 4 shows that a trimethylated K79-specific histone H3 antibody detected a signal after Ddc1 and Ddc2 induction in *Δelg1* and *Δelg1 Δddc1* cells that was indistinguishable from that seen with the WT cells.

10.1128/mBio.01159-19.5 TABLE S2 List of primers. Download Table S2, DOCX file, 0.01 MB. Copyright © 2019 Sau et al. 2019 Sau et al. This content is distributed under the terms of the Creative Commons Attribution 4.0 International license.

To detect the recruitment of Rad9 and Dpb11 at the LacO array, we performed a chromatin immunoprecipitation (ChIP) assay with G_2_-M-arrested WT and *Δelg1* cells following galactose induction to colocalize Mec1-Ddc2 and 9-1-1. Panels B and C of Fig. 4 show that Rad9 and Dpb11 were recruited to the LacO arrays at similar levels in the *Δelg1* and WT cells. It is also clear that Rad9 and Dpb11 were recruited specifically at the Lac operator sites, as we did not detect their recruitment at the nonspecific *ADE2* site.

### Activation of Rad9 and Dpb11 requires Elg1.

Next, we asked whether phosphorylation of Rad9 and/or Dpb11 might be defective in *elg1* strains. To carry out Rad9 and Dpb11 phosphorylation assays, we placed 5×-FLAG epitopes at the C terminus of Rad9 and a Myc epitope at the C terminus of Dpb11. We then performed a G_2_-M arrest checkpoint induction assay as described in the Fig. 2B legend and used anti-FLAG and anti-Myc antibodies to detect the tagged proteins. In the WT strain, Rad9 became phosphorylated following the induction, whereas no phosphorylation was observed in the *Δelg1* strain (Fig. 4D). A similar result was observed for Dpb11 (Fig. 4D). We found earlier that a functionally compromised *elg1* mutant (*elg1-sim+386/7DD*) and a phospho-deficient *elg1* mutant (*elg1-S112A*) displayed defects in activating Rad53 kinase (Fig. 3B). Similarly, we found that both these *elg1* derivatives were unable to phosphorylate Rad9 (Fig. S2). These results demonstrate that Elg1 is required for Rad9 and Dpb11 phosphorylation and possibly explain why the Rad53 kinase stays inactive in *elg1* mutants, as modification of the adaptor proteins is required for Rad53 phosphorylation.

10.1128/mBio.01159-19.2 FIG S2 Absence of Rad9 phosphorylation in functionally compromised as well as in phosphorylation-deficient Elg1 cells. The phosphorylation levels of Rad9 in WT, *Δelg1*, *elg1-SIM+DD*, and *elg1-S112A* cells are indicated. Actin was used as a loading control. Download FIG S2, TIF file, 0.3 MB. Copyright © 2019 Sau et al. 2019 Sau et al. This content is distributed under the terms of the Creative Commons Attribution 4.0 International license.

### Exo1 is not required for Elg1-mediated DNA damage checkpoint activation.

**FIG 5:**
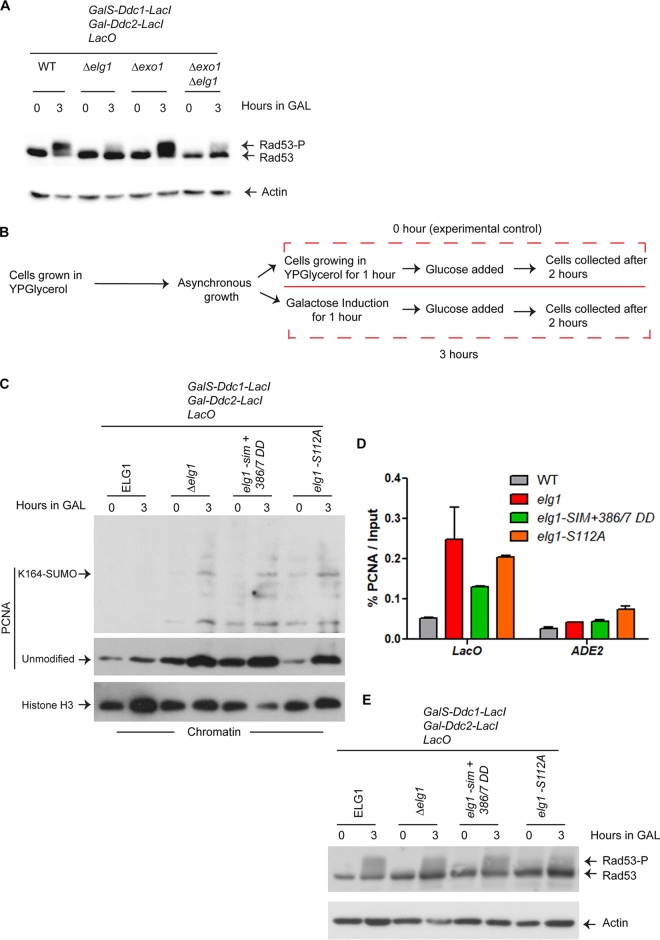
Accumulated Lac operator site-PCNA impedes Rad53 phosphorylation. (A) Exo1 is not required to activate Rad53 in the DNA damage checkpoint. The checkpoint induction experiments were carried out with WT, *Δelg1*, *Δexo1*, *Δexo1 Δelg1* to detect phosphorylation of Rad53. Actin was used as a loading control. (B) The schematic diagram represents the experimental regime for Fig. 5C. C. A Western blot of chromatin fractionation assay-samples detects the PCNA level in the indicated strains (*ELG1*, *Δelg1*, *elg1-sim + 386/7 DD*, and *elg1-S112A*). The histone H3 was used as a chromatin-bound control and also served as a loading control. (D) The checkpoint induction experiments, as described in Fig. 2B, were carried out with Fig. 5C cells, and PCNA was pulled down using anti-PCNA antibody. The histograms of chromatin immunoprecipitated PCNA represents the accumulation level of PCNA at the LacO array in WT and *elg1* mutants. *ADE2* amplification was the experimental control. At least three biological repeats were carried out; error bars represent standard errors of the means (SEM). One-way ANOVA was performed to obtain *p* values, and Tukey’s multiple-comparison test was carried out to find statistical significance between groups. *P = *0.0344; *, *P* < 0.05. (E) The checkpoints were induced in the same set of cells (i.e., those used as described in the panel C and D legends) after arresting them at G_1_, and Rad53 phosphorylation levels were detected. Actin was used as a loading control.

Exo1 is a 5′-to-3′ DNA exonuclease with an important role in mismatch repair and processing of double-stranded breaks. In a recent work, Ulrich and colleagues showed that Exo1 plays an important role in Rad9-dependent Rad53 activation induced by the presence of single-stranded gaps behind a replication fork (13). To explore a possible role of Exo1 in the Elg1-mediated checkpoint activation, we created *Δexo1* and *Δexo1 Δelg1* mutants. The *Δexo1* mutant showed Rad53 phosphorylation at a level similar to that shown by the WT strain, whereas the *Δexo1 Δelg1* double mutant failed to phosphorylate Rad53 and behaved similarly to the *Δelg1* strain (Fig. 5A). This result shows that Exo1 does not mediate the Elg1-dependent elicitation of the DNA damage checkpoint.

### Accumulated PCNA at Lac operator sites blocks Rad53 activation.

We have thus far shown that Rad53 kinase remains inactive in the absence of Elg1 or in the presence of functionally compromised or phospho-deficient Elg1 (Fig. 3B). In all these cases, the primary reason for an inactive checkpoint response might be the accumulated chromatin-bound PCNA levels, as the *elg1-sim+386/7DD* and *elg1-S112A* strains, similarly to the *Δelg1* strain, fail to unload chromatin-bound PCNA under DNA damage conditions (41, 42) (Fig. 1). To test this notion, we carried out a chromatin fractionation assay of asynchronous WT and *elg1* mutants as described for Fig. 5B. It is clear from the data presented in Fig. 5C that all of the *elg1* mutants (*Δelg1*, *elg1-sim+386/7DD*, and *elg1-S112A*) accumulated both unmodified PCNA and SUMOylated PCNA at 3 h compared to 0 h whereas PCNA was efficiently removed from WT cells. Chromatin immunoprecipitation of PCNA at the LacO array with G_2_-M-arrested WT and *elg1* mutants following galactose induction (Fig. 5D) confirmed that all of the *elg1* mutants accumulated significantly higher levels of PCNA at the LacO arrays upon induction than were seen with the WT cells.

The results described above suggest that the PCNA that accumulated at the Lac operator sites in *elg1* mutants blocked the phosphorylation of Rad53. It has been reported that in the absence of Elg1, PCNA accumulates on chromatin during the S phase and beyond but not in the G_1_ phase (32). Thus, if LacO-bound PCNA blocks Rad53 phosphorylation, G_1_-arrested *elg1* mutants, which do not accumulate PCNA, should be able to phosphorylate Rad53. Panel E of Fig. 5 clearly shows that this is the case, although the level of Rad53 phosphorylation by Ddc1/Ddc2 induction is lower in G_1_-arrested cells than in G2-arrested cells in which the cyclin-dependent kinase (CDK) is fully active, as expected (20, 58). These results thus confirm that the accumulation of PCNA in the absence of proper Elg1 function prevents the activation of the DNA damage checkpoint.

## DISCUSSION

**FIG 6:**
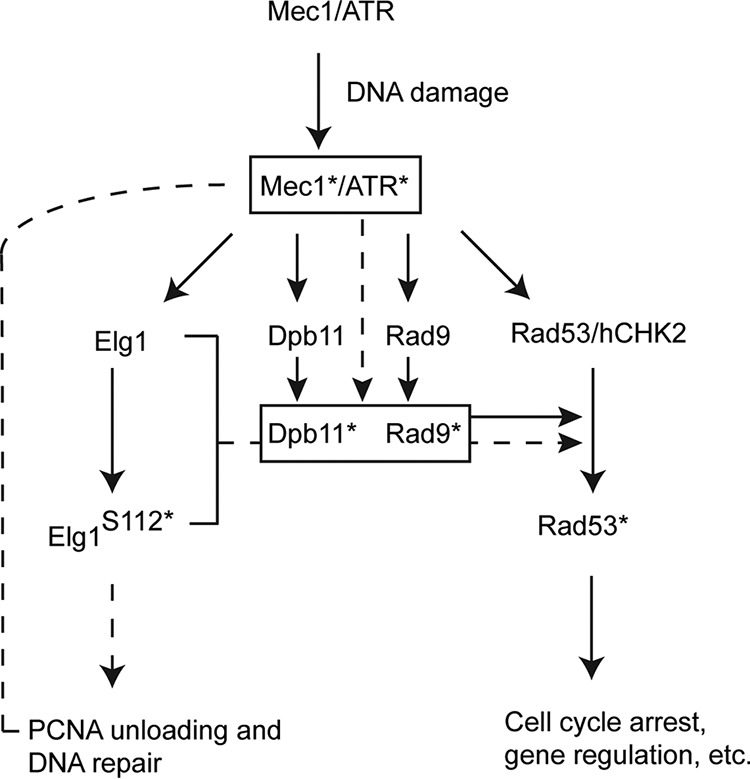
A model for the potential role of Elg1 in the activation of the DNA damage checkpoint. The current results are consistent with the following scenario: in response to DNA damage, Mec1 becomes activated (Mec1*). This, in turn, results in the phosphorylation of Rad53 (Rad53*) as well as serine 112 of Elg1 (Elg1 S112*). Phosphorylated Elg1 helps in the DNA repair process by unloading the chromatin-bound PCNA during DNA damage, whereas phosphorylated Rad53 transduces the signal to complete checkpoint functions related to cell cycle arrest. The checkpoint sensor (Dpb11) and mediator (Rad9) are phosphorylated (Dpb11* and Rad9*, respectively) by Mec1*, which in turn generates Rad53* through Dpb11*/Rad9*. The chromatin-bound PCNA unloading activity of Elg1 is necessary for Rad53 phosphorylation as absence of this step resulted in a failure to phosphorylate both Dpb11 and Rad9. The accumulated PCNA at the damaged site (here, the Lac operator site) probably controls the Dpb11*/Rad9* formation by blocking Mec1* activity (see details in Discussion) and hence controls the Rad53 kinase activity. Solid lines denote findings from previously published work, and dashed lines indicate the findings of the present study.

The Elg1 RFC-like complex plays an important role as an unloader of PCNA during DNA replication (35). Failure to unload PCNA beyond the S phase leads to DNA sensitivity and genomic instability (42, 59). Here we show that Elg1 also plays a central role in the activation of the DNA damage checkpoint. As summarized in Fig. 6, our results show that PCNA unloading by Elg1 from damaged chromatin sites is required for Rad53 activation.

We showed previously that following DNA damage, Mec1^ATR^ phosphorylates Elg1 at serine 112, which forms a Mec1^ATR^/Tel1^ATM^-preferred SQ motif (41). Here, using S112 mutants, we show that Mec1-dependent Elg1 phosphorylation is not required for chromatin-bound PCNA unloading during the regular S phase but plays a significant role under DNA damage conditions (Fig. 1). Whereas mutation in S112 to alanine (S112A) mimics an unphosphorylated state, mutations to glutamic acid (S112E) sometimes mimic a phosphorylated state. However, the two mutants showed the same lack of checkpoint induction and the same high level of PCNA accumulation (Fig. 1). Either the S112E allele fails to mimic a true phosphorylation event or serine 112 may play a structural role in addition to being a site of phosphorylation. We also note that S112 is located close to SUMO interaction motif 3 (SIM 3; *116EDDISII122*), which is necessary for proper PCNA unloading after DNA damage (42). Phosphorylation of this site may thus play a structural role in the interactions with PCNA after DNA damage.

By using inducible checkpoint-bearing strains that elicit the DDR in the absence of actual DNA damage (47, 48), we have shown that the replication checkpoint was functional in the absence of Elg1, whereas the DNA damage branch was defective (Fig. 2).

Phosphorylation of Rad53 requires the presence of phosphorylated Rad9 and Dpb11 at the damaged site (55). We found that the levels of Dot1-mediated lysine 79 methylated histone H3 and of Mec1/Tel-mediated phosphorylated S129 of histone H2A (γ-H2A), two factors required for Rad9 recruitment to damaged sites, were unaffected by the absence of Elg1 (Fig. 2 and 4). Moreover, both Rad9 and Dpb11 were recruited normally to the Lac operator sites in the absence of Elg1. However, both proteins failed to be phosphorylated, and thus, the initial checkpoint signal was not amplified (Fig. 4). What is the reason for such a failure? Cdc28, the yeast CDK, phosphorylates Rad9 at residues S462 and T474, allowing its recruitment alongside Dpb11 at damaged sites (20). This stage of the activation appears normal, as the two proteins are recruited to the LacO arrays similarly in *Δelg1* and WT cells (Fig. 4). The full activation of Rad9, however, depends on the phosphorylation of an S/TQ cluster domain (SCD) by Mec1/Tel1 (60), which in turn provides a docking surface for the forkhead-associated domains (FHD) of Rad53, needed for its phosphorylation and activation (61). As the primary function of Elg1 is that of unloading the chromatin-bound PCNA, we suggest that Rad9 activation by Mec1 may require the unloading of PCNA from the damaged chromatin sites, either because the PCNA molecules physically perturb the reaction or because PCNA unloading generates an essential signal required for Mec1 to fully phosphorylate Rad9 and Dpb11 and thereby to elicit the checkpoint response. In accordance with this conclusion, we found that PCNA accumulated at the damaged chromatin sites (here at the Lac operator sites) in *elg1* mutants and that the checkpoint activation was not affected by mutation of Elg1 in G_1_-arrested cells (Fig. 5).

Our study uncovered a mechanism that links to PCNA unloading during checkpoint activation. Upon DNA damage, Mec1 activation leads to Elg1 phosphorylation, required for proper PCNA unloading. This step is in turn necessary for Rad9/Dpb11 phosphorylation, which controls Rad53 phosphorylation by Mec1. It is possible that Elg1phosphorylation alters the interaction with other proteins or affects the efficiency of PCNA unloading. Our results show that, whereas Mec1/Rad53 controls PCNA unloading to facilitate repair, reciprocally, Elg1-RLC activity controls Mec1/Rad53 kinase activation.

## MATERIALS AND METHODS

### Yeast strains.

All yeast strains used in this study were derivatives of MK166 (62) and/or W303 (63) and are listed in Table S1 in the supplemental material. Reported mutants, and strains carrying a specific epitope tag at their C termini, were created using standard yeast manipulation techniques.

10.1128/mBio.01159-19.4 TABLE S1 Yeast strains. Download Table S1, DOCX file, 0.01 MB. Copyright © 2019 Sau et al. 2019 Sau et al. This content is distributed under the terms of the Creative Commons Attribution 4.0 International license.

### Cell growth and cell cycle arrest.

Yeast cells were grown at 30°C in yeast extract-peptone (YP) medium supplemented with either dextrose or glycerol/galactose as required or as mentioned in the text or figure legends. For experiments investigating checkpoint-inducible systems, cells were arrested at G_2_-M using the microtubule depolymerizing drug nocodazole (15 μg/ml) for 90 min. For G_1_ arrest experiments, cells were treated with alpha-factor and kept arrested in G_1_ for the duration of the experiment.

### Chromatin fractionation assay.

The chromatin isolation protocol was followed as reported previously (42) with minimal modifications. For fractionation of nuclei and cytoplasm, equal amounts of protein from each sample were layered over 1 ml NIB (20 mM Tris-Cl [pH 7.4], 100 mM NaCl, 1.2 M sucrose, 10 mM dithiothreitol [DTT], and appropriate protease inhibitors) and centrifuged at 12,000 × *g* for 15 min at 4°C. The glassy white nuclear pellet was resuspended in 500 μl EBX buffer (20 mM Tris.Cl pH 7.4, 100 mM NaCl, 1% Triton X-100, and appropriate protease inhibitors). Triton X-100 was added to reach a 1% final concentration to lyse the nuclear membrane, and the samples were kept on ice for 10 min with gentle mixing. The chromatin was collected by centrifugation (15,000 × *g*, 10 min, 4°C). Chromatin was resuspended in 80 μl of EBX buffer, and 20 μl of 5× SDS-PAGE loading buffer (250 mM Tris Cl [pH 6.8], 0.05 M DTT, 10% SDS, 50% glycerol, 0.5% bromophenol blue) was added. The samples were placed in a boiling water bath for 3 to 4 min and then sonicated six times for 10 s each time at 80% amplitude (Sonics; Vibra Cell, USA) with at least 1 min on ice between pulses. The samples were loaded on 12% and 15% SDS-polyacrylamide gels to probe PCNA and histone H3, respectively, through Western blotting.

### Protein extraction, Western blotting, antibodies, and quantification.

Proteins were extracted from cells as described previously using either a trichloroacetic acid method (64) or a spheroplast method (42). To resolve Rad9, Rad53, and actin, 6% to 10% SDS-polyacrylamide gels were used. To detect Dpb11 phosphorylation, 6% Phos-tag SDS-polyacrylamide gel was used. Immunoblotting was done as described previously (64).

To detect proteins and/or proteins with specific epitopes, the following primary antibodies were used: mouse anti-PCNA (ab70472) (1:1,000), mouse anti-actin (MP catalog no. 08691001) (1:1,000), rabbit anti-histone H3 (ab1791) (1:5,000), rabbit anti-histone H2A (phospho-S129) (ab15083) (1:1,000), rabbit anti-histone H3 (trimethyl K79) (ab2621) (1:1,000), mouse anti-FLAG (F1804-Sigma) (1:1,000), mouse anti-Myc (sc-40) (1:1,000), and goat anti-Rad53 (sc-6749) (1:1,000). The following secondary antibodies (from Jackson ImmunoResearch Laboratories, Inc.) were used: goat anti-mouse horseradish peroxidase (HRP) (ab97040) (1:5,000), goat anti-rabbit HRP (ab97080) (1:5,000), and donkey anti-goat HRP (1:5,000).

Protein intensity was quantified from immunoblots using ImageJ software (NIH, USA).

### Chromatin immunoprecipitation (ChIP)-qPCR assay.

ChIP assays were carried out as described previously (64, 65). For quantitative PCR (qPCR), a StepOnePlus real-time PCR system (Thermo Fisher Scientific, USA) was used. For a 20-μl SYBR green reaction mixture, 6 μl of IP DNA and 6 μl of 1:100 diluted input DNA were used. The qPCR data were calculated using the percent input method (Thermo Fisher Scientific, USA). Data from all ChIP experiments are plotted as averages of results from at least three independent biological repeats (each biological repeat represented the average of the results from three technical PCR repeats). The *LacO* and *ADE2* primer sequences are listed in Table S2.
