# Supplementary figures and images for: The Yeast PCNA Unloader Elg1 RFC-Like Complex Plays a Role in Eliciting the DNA Damage Checkpoint
Source: mBio. 2019 Jun 11;10(3):e01159-19. doi: 10.1128/mBio.01159-19 (PMC6561032; doi:10.1128/mBio.01159-19)

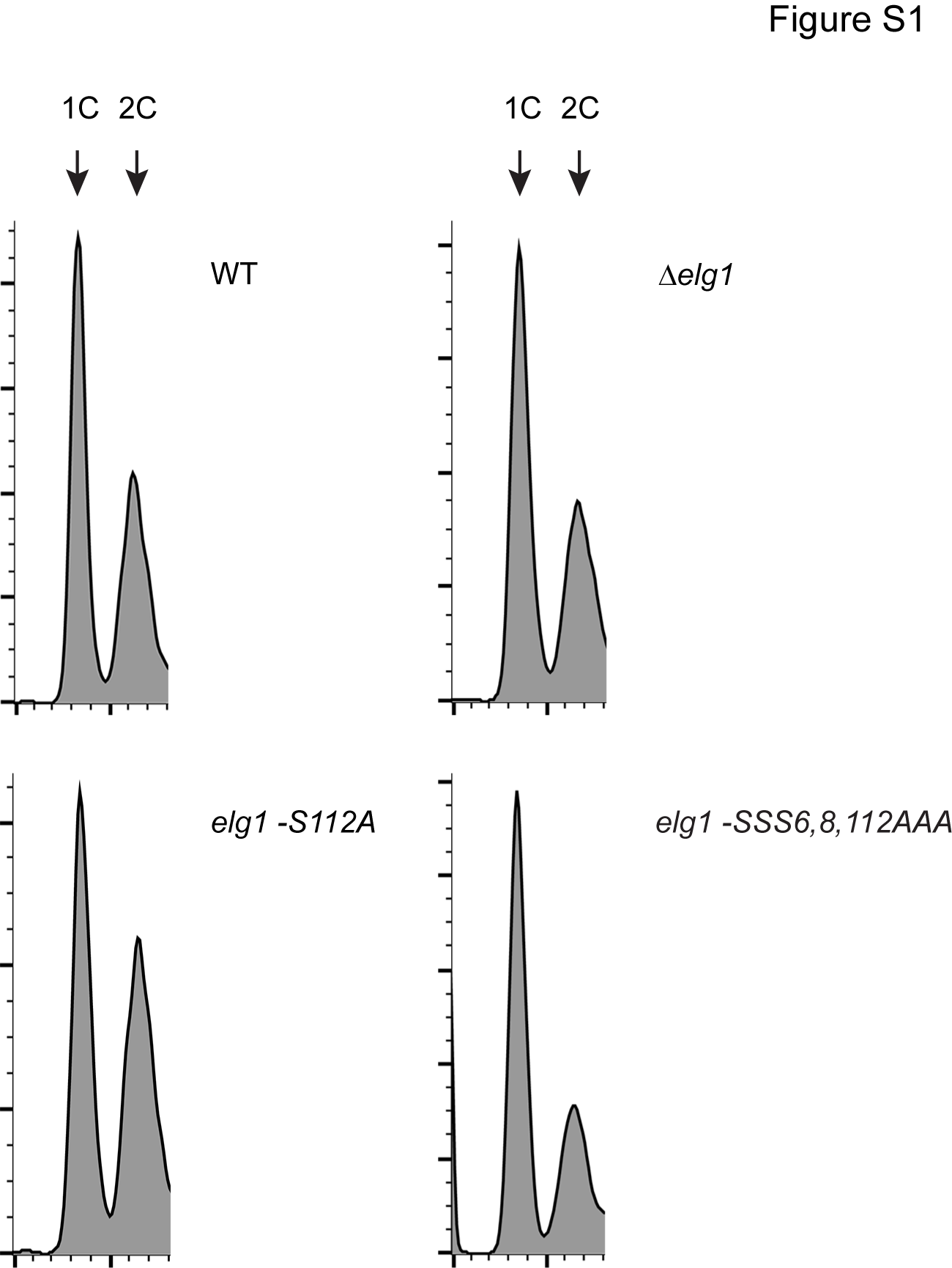

Supplement: FIG S1 [file mBio.01159-19-sf001.tif]

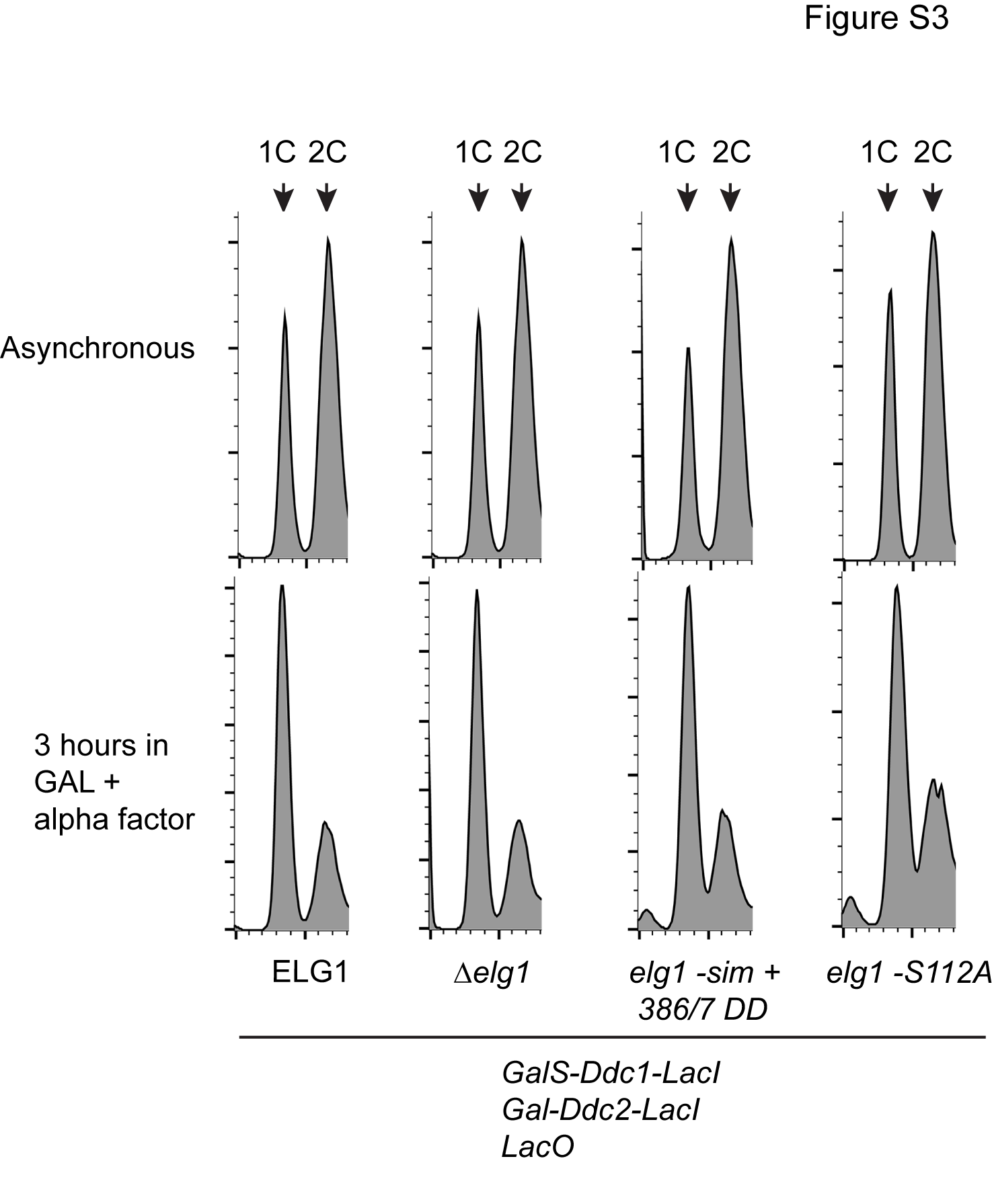

Supplement: FIG S3 [file mBio.01159-19-sf003.tif]

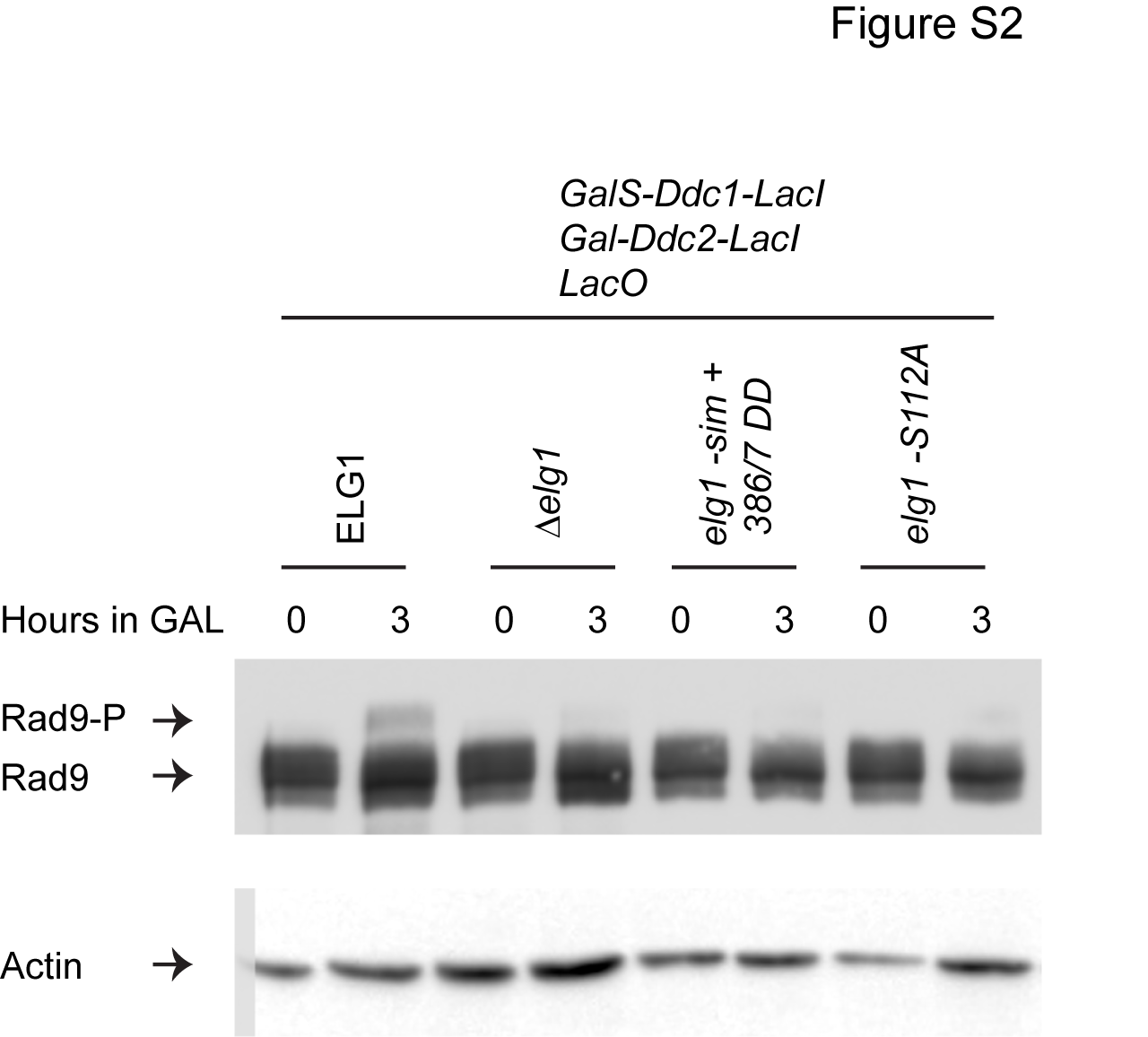

Supplement: FIG S2 [file mBio.01159-19-sf002.tif]
