# Supplementary material for: The Yeast PCNA Unloader Elg1 RFC-Like Complex Plays a Role in Eliciting the DNA Damage Checkpoint
Source: mBio. 2019 Jun 11;10(3):e01159-19. doi: 10.1128/mBio.01159-19 (PMC6561032; doi:10.1128/mBio.01159-19)
Supplement: TABLE S2 [file mBio.01159-19-st002.docx]

Table S2: List of primers

| Oligo description | Sequence | Source |
| --- | --- | --- |
| LacO ChIP Fwd.  LacO ChIP Rev.  *ADE2* ChIP Fwd.  *ADE2* ChIP Rev. | TGGAATTCTCGAGGGATCCCC  CTGCAAGGCGATTAAGTTGGG  GGCGGAATGTGAACAAAGGCTGAAC  GGCAGACATTACCGGCAAGTC | (54)  (54)  This study  This study |
