# Supplementary material for: The Yeast PCNA Unloader Elg1 RFC-Like Complex Plays a Role in Eliciting the DNA Damage Checkpoint
Source: mBio. 2019 Jun 11;10(3):e01159-19. doi: 10.1128/mBio.01159-19 (PMC6561032; doi:10.1128/mBio.01159-19)
Supplement: TABLE S1 [file mBio.01159-19-st001.docx]

Table S1: Yeast strains

| Strain Name | Genotype | Source |
| --- | --- | --- |
| MK166 | Mat a *lys2:: TySup ade2-1(0c) can1-100 (0c) ura3-52 leu2-3,112 trp1Δ901 HIS3:: lys2:: ura3- his4::TRP1::his4* | (35) |
| MK14760 | MK166 *Δelg1::HygMXR* | (32) |
| MK14593 | MK166 *elg1-SSS6,8,112AAA::KanMX^R^* | (29) |
| MK14724 | MK166 *elg1-S112A::KanMX^R^* | (29) |
| MK14599 | MK166 *elg1-SSS6,8,112EEE::KanMX^R^* | (29) |
| MK14727 | MK166 *elg1-S112E::KanMX^R^* | (29) |
| CBY88 | Mat a *ade2-1 Gal-Ddc2-LacI::HIS3 Rad53-HA::LEU2 LacO256::TRP1 GalS-Ddc1-LacI::URA3 ddc1∆* | (39) |
| SS123 | CBY88 *Δelg1::HygMX^R^* | This study |
| TBY79 | Mat a *GalS-Mrc1-LacI::ADE2 Gal-Ddc2-LacI::HIS3 Rad53-HA::LEU2 LacO180::TRP1 ura3-1* | (40) |
| SS127 | TBY79 *Δelg1::HygMX^R^* | This study |
| SS168 | Mat a *ade2-1 Gal-Ddc2-LacI::HIS3 Rad53-HA::LEU2 LacO256::TRP1 GalS-Ddc1-LacI::URA3* | This study |
| SS171 | Mat a *ade2-1 Gal-Ddc2-LacI::HIS3 Rad53-HA::LEU2 LacO256::TRP1 GalS-Ddc1-LacI::URA3 Δelg1::HygMX^R^* | This study |
| MK16896 | CBY88 *Δctf18::KanMX^R^* | This study |
| MK16895 | TBY79 *Δctf18::KanMX^R^* | This study |
| SS267 | CBY88 *elg1-sim+386/7DD::KanMX^R^* | This study |
| SS275 | CBY88 *elg1-S112A::KanMX^R^* | This study |
| SS294 | CBY88 *Rad9-5xFLAG::HygMX^R^* | This study |
| SS296 | CBY88 *Δelg1::NatMX^R^* *Rad9-5xFLAG::HygMX^R^* | This study |
| SS223 | Mat a *ade2-1 Gal-Ddc2-LacI::HIS3 Rad53-HA::LEU2 LacO256::TRP1 GalS-Ddc1-LacI::URA3 Rad9-5xFLAG::HygMX^R^* *Dpb11-13MYC::KanMX^R^* | This study |
| SS208 | Mat a *ade2-1 Gal-Ddc2-LacI::HIS3 Rad53-HA::LEU2 LacO256::TRP1 GalS-Ddc1-LacI::URA3 Δelg1::NatMX^R^* *Rad9-5xFLAG::HygMX^R^* *Dpb11-13MYC::KanMX^R^* | This study |
| SS305 | CBY88 *Δexo1::KanMX^R^* | This study |
| SS307 | CBY88 *Δelg1::HygMX^R^* *Δexo1::KanMX^R^* | This study |
| MK17549 | MK166 *Δsml1::KanMX^R^* | This study |
| MK17552 | MK166 *Δsml1::KanMX^R^* *Δmec1::HygMX^R^* | This study |
| MK17553 | MK166 *Δsml1::KanMX^R^* *Δmec1::HygMX^R^ Δelg1::URA3* | This study |
| MK17555 | MK166 *Δsml1::KanMX^R^* *Δrad53::HygMX^R^* | This study |
| MK17557 | MK166 *Δsml1::KanMX^R^* *Δrad53::HygMX^R^ Δelg1::URA3* | This study |
